# Simulating local adaptation to climate of forest trees with a Physio-Demo-Genetics model

**DOI:** 10.1111/eva.12143

**Published:** 2014-02-21

**Authors:** Sylvie Oddou-Muratorio, Hendrik Davi

**Affiliations:** INRA, UR629 Ecologie des Forêts Méditerranéennes (URFM)Avignon, France

**Keywords:** budburst phenology, ecophysiology, European beech, *Fagus sylvatica*, individual-based model, quantitative genetics, quantitative trait loci

## Abstract

One challenge of evolutionary ecology is to predict the rate and mechanisms of population adaptation to environmental variations. The variations in most life history traits are shaped both by individual genotypic and by environmental variation. Forest trees exhibit high levels of genetic diversity, large population sizes, and gene flow, and they also show a high level of plasticity for life history traits. We developed a new Physio-Demo-Genetics model (denoted PDG) coupling (i) a physiological module simulating individual tree responses to the environment; (ii) a demographic module simulating tree survival, reproduction, and pollen and seed dispersal; and (iii) a quantitative genetics module controlling the heritability of key life history traits. We used this model to investigate the plastic and genetic components of the variations in the timing of budburst (TBB) along an elevational gradient of *Fagus sylvatica* (the European beech). We used a repeated 5 years climatic sequence to show that five generations of natural selection were sufficient to develop nonmonotonic genetic differentiation in the TBB along the local climatic gradient but also that plastic variation among different elevations and years was higher than genetic variation. PDG complements theoretical models and provides testable predictions to understand the adaptive potential of tree populations.

## Introduction

The ongoing and predicted rapid changes in temperature, precipitation and CO_2_ atmospheric concentration, and the resulting increase in the frequency and intensity of extreme events such as droughts will have a wide range of long-term implications for natural population dynamics and ecosystem sustainability. Within a population, these changes impose strong selective pressures, which affect demographic rates and can cause genetic evolution across generations (Hansen et al. 2012). Moreover, climate change (CC) also affects the physiology and development of individual organisms up to the limits of their phenotypic plasticity, that is, the ability of individual genotypes to produce alternative phenotypes in different environments (Chevin et al. 2013). The interplay between genetic evolution and phenotypic plasticity ultimately determines a population's ability to adjust (without migrating) to novel environmental conditions imposed by CC. Investigating these mechanisms is essential for predicting eco-evolutionary dynamics and ecosystem services and for guiding conservation efforts.

This issue is crucial for trees because of their pivotal role in the functioning and biodiversity of forest ecosystems. Multisite experiments (using forester provenance tests) showed that current tree populations can adjust to varying environmental conditions through phenotypic plasticity over a non-negligible latitudinal range (Rehfeldt et al. 2002). Moreover, based on the patterns of local adaptation displayed by most tree species over the course of postglacial recolonization, forest tree populations are usually assumed to have a high evolutionary potential (Savolainen et al. 2007; Alberto et al. 2013); however, tree population abilities of genetic evolution over a short timescale (i.e., microevolution) remain largely unresolved. In addition, plasticity and genetic adaptation can interact together and with gene flow, as illustrated mostly by theoretical models or studies of model species. One well-known interaction is the interplay between gene flow and adaptation when the environment changes both in space and in time (Polechová et al. 2009). Trees are capable of long-distance pollen-mediated gene flow, which could promote adaptive evolution to novel environments (Kremer et al. 2012). Another pervasive interaction involves plasticity and genetic adaptation; plasticity can be adaptive if plastic trait variation increases individual fitness (Nicotra et al. 2010), or it can be maladaptive if plasticity decreases fitness (Ghalambor et al. 2007). Moreover, when adaptive plasticity cannot evolve, it can slow down the genetic response to directional selection, but it also allows phenotypes to track environmental change more closely (Chevin et al. 2013).

Methodological developments currently limit our understanding of the interplay among plasticity, genetic adaptation, and gene flow and their impacts on tree population dynamics. In most evolutionary models thus far developed for tree life history traits, individual fitness is either directly controlled by the genotype (Le Corre and Kremer 2003) or derived from genetically controlled life history traits (Kuparinen et al. 2010). Climate effects on water and carbon exchanges are a complex process that has been studied by ecophysiologists and have rarely been explicitly taken into account as a selective pressure in evolutionary models (but see (Kramer et al. 2008). Similarly, the interindividual variation and adaptive potential of traits related to climate response have rarely been incorporated into biophysical and ecophysiological models.

The different time scales considered by ecophysiological and evolutionary models (typically from the hour to the year or tens of years for the former versus many generations at equilibrium for the latter) are generally considered to be challenges in the development of coupled physio-demo-genetic models. However, neither of these time scales may be relevant for forming accurate predictions of realistic tree population responses to CC. Indeed, current forest tree populations can rarely be considered to be at equilibrium, and demographic processes play a major role in the dynamics of adaption over a few generations (Savolainen et al. 2007). Moreover, CC is likely to involve noncontinuous and nonpredictable change in response to abiotic conditions, which limit the relevance of long-term predictions at equilibrium (Kremer and Le Corre 2011). In contrast, the predictions of biophysical and ecophysiological models cannot be generalized over more than one generation if the microevolution of functional traits within a population is not negligible. Therefore, there is a need for physio-demo-genetic models to address the timescale of a few generations (<10).

In this study, we propose a new Physio-Demo-Genetics model (PDG) coupling the following: (i) a functional module derived from CASTANEA (Dufrêne et al. 2005) to simulate carbohydrate and water fluxes at the tree level using daily climate observations; (ii) a population dynamics module to convert carbohydrate reserves into demographic rates for adult trees (growth, mortality, and seed production) and to simulate ecological processes across the life cycle (including seed and pollen dispersal, germination rate, and density-dependent mortality of seedlings); and (iii) a quantitative genetics module relating genotype of the quantitative trait loci (QTL) to the phenotype of one or more functional traits (Labonne and Hendry 2010). This individual-based, spatially explicit model simulates the evolution of functional traits in tree populations, where phenotypic differences between individuals are determined by their genotype at QTLs that control functional traits and by their physiological response to local climate conditions. PDG is available from the CAPSIS modeling platform (Dufour-Kowalski et al. 2012).

We used PDG to simulate local adaptation in a continuous tree population that expands along an elevational gradient based on experimental data collected from *Fagus sylvatica* populations on Mont Ventoux in southeastern France. Under divergent selection, local adaptation is expected to result in phenotypic differentiation for traits contributing to fitness across the gradient. Among the various traits contributing to fitness, we focused on the timing of budburst (TBB), a phenological trait that determines the length of the growing season in *F. sylvatica* (Davi et al. 2011). An earlier budburst extends the time during which photosynthesis occurs (Richardson et al. 2006), but it also increases the risk of late frost damage (Dittmar and Elling 2006). *In situ* observations of *F. sylvatica* on Mont Ventoux revealed a classical phenotypic cline in TBB resulting from plastic variation; budburst occurs earlier at lower than higher elevations because TBB is triggered by the heat sum (Davi et al. 2011). The opposite is observed for genetic clines as assessed in common garden experiments, where TBB is observed under the same environmental conditions for all populations; under such conditions, *F. sylvatica* populations originating from higher elevations show earlier budburst than those originating from lower elevations (von Wuehlisch et al. 1995; Vitasse et al. 2009a; Gomory and Paule 2011). This situation in which the phenotypic and genetic clines vary in opposite directions is referred to as a counter-gradient variation. In contrast, genetic and phenotypic clines have been shown to exhibit co-gradient variation for TBB in some species (e.g., *Quercus* sp.), while clear linear genetic clines are not observed for other species (e.g., *Fraxinus*) (Vitasse et al. 2009a).

In this article, we illustrate the potential of PDG to elucidate the processes through which adaptation proceeds for *F. sylvatica* on Mont Ventoux. We address the following issues: (i) How do adaptive genetic variation and phenotypic plasticity contribute to TBB variation along an elevational gradient? (ii) How fast can genetic differentiation in TBB develop? and (iii) Is there a monotonic trend in the genetic variation across the gradient?

## Materials and methods

### Overview of PDG model

#### The physiological process-based module

This module corresponds to the CASTANEA library hosted on the CAPSIS platform. Initially developed at the stand scale, CASTANEA (Dufrêne et al. 2005) simulates canopy photosynthesis (i.e., gross primary production, GPP) and transpiration, maintenance and growth respiration, seasonal development and assimilate partitioning to leaves, carbohydrate storage (hereafter reserves), stems, branches, and coarse and fine roots. The meteorological driving variables are global radiation, rainfall, wind speed, air humidity, and temperature. A complete description of the model is given in Dufrêne et al. (2005), and the submodel of carbon allocation is described by Davi et al. (2009).

In its initial version, CASTANEA simulated CO_2_ and H_2_O fluxes, considering one average tree as representative of the whole stand. To account for interindividual variation, we considered each tree as a single unit with its own parameters for the CASTANEA simulation. Note that all CASTANEA units were treated independently, meaning that we do not account for competition among trees for light or soil water acquisition. In contrast to the stand-level version, we computed several variables at the individual tree level, including biomass (*B*_tree_), leaf area index (LAI) by tree, and crown projection (*A*_crown_), to determine the carbon and water budgets of each tree (see Appendix S1).

Only one of all the CASTANEA parameters was allowed to vary among trees, namely, *F*_critBB_, the critical value of the state of forcing, which is most commonly referred to as the temperature sum required for budburst. The TBB was simulated following the equs 1–3:


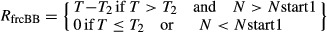
(1)

where *R*_frcBB_ is the rate of forcing for bud break, T the mean daily temperature, *T*_2_ the base temperature, *N* the day of year, and *N*_START1_ the date of onset of rest.


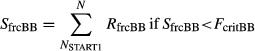
(2)


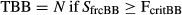
(3)

with *S*_frcBB_ the state of forcing, *F*_critBB_ the critical value of state of forcing for the transition from quiescence to the active period and TBB the day when bud break occurred.

We chose to focus on the *F*_critBB_ variation for two reasons. First, *F*_critBB_ is one of 17 key parameters controling carbon exchange according to the sensitivity analyses performed in Dufrêne et al. (2005). Second, the genetic clines observed for TBB in a common garden of populations from different elevations are likely to result from among-population variation for *F*_critBB_. For the sake of simplicity, we assumed that the effect of chilling on dormancy was constant across year and elevations.

To assess the impact of drought on photosynthesis, an annual water stress index (WSI) was estimated at the tree level as the sum of the daily reduction of photosynthesis caused by soil drought (i.e., when the relative soil water content drops below 40% of the soil water reserve).

In this version of CASTANEA, we also added the effect of late frost on LAI. Trees are most sensitive to frost during budburst; frost can kill the new shoots, reduce growth, and cause misshapen branching (Dittmar and Elling 2006). When unfolding leaves are affected by frost, decreased leaf area is expected. In PDG, every day during which the minimal daily temperature (*T*_min_) fell below a threshold value (*T*_minEffect_) following the initiation of budburst was considered to affect the LAI as follows:


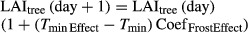
(4)

##### Parameterization

**Figure 1:**
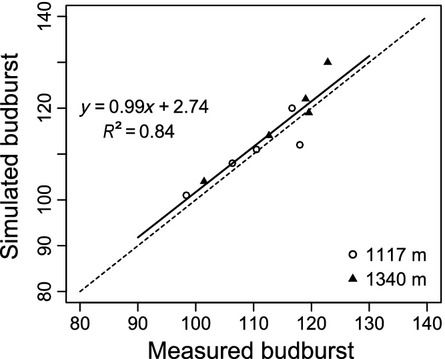
Observed budburst in Mont Ventoux versus simulated budburst using an average value of 190°C for the temperature sum required for budburst (*F*_critBB_).

In previous studies, species-specific CASTANEA parameters for *F. sylvatica* were determined and CASTANEA was validated at an experimental site in Northern France (Davi et al. 2005). Additionally, some site-specific parameters were measured in Ventoux (Table 1). First, the budburst model was calibrated using two types of data. The onset date of rest (*R*_frcBB_) was estimated by an experiment on dormancy release in the spring of 2012 (unpublished data). The average critical value for the state of forcing (*F*crit_BB_) was estimated using budburst survey from 2007 to 2011 at two elevations (1117 and 1340 m) for 20 trees per elevation (Fig. 1). Characteristics of sun leaves (nitrogen content and LMA = leaf mass per area) were obtained for 149 trees in an intensive-studied plot (Bontemps 2012). Canopy clumping (CI) was estimated using five hemispherical photographs taken in the same plot in the summer of 2008, following the methodology described in Davi et al. (2008). The photosynthesis parameter (maximum carboxylation rate = 51.6 μmol photon m^−2^ s^−1^) was estimated from previous measurements at the same site in the summer of 2006 (M. Ducrey and R. Huc, personal communications).

**Table 1:** List of Physio-Demo-Genetics parameters.

Parameter	Acronym	Value	Unit	Sources
Physical/physiological CASTANEA module				
Canopy clumping		0.56	–	Davi et al. (2008)
Nitrogen content		2.2	%	Bontemps (2012)
Leaf mass per area		93	g_DM_ m^−2^	Bontemps (2012)
Relationship between maximal rate of carboxylation and nitrogen		26.04	μmol CO_2_ g N^−1^ s^−1^	Ducrey and Huc (personal communications)
Date of rest onset for budburst		78	Days	Davi (unpublished data)
Average critical value of forcing state	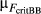	190	°C	This study
Base temperature for forcing budburst		0	°C	Fixed
Ratio between fine root and leaf biomass		1	–	Fixed
Soil extractable water	SEW	60	mm	Nourtier et al. (2012)
Threshold value for frost effect on leaf area index	*T*_minEffect_	0	°C	Fixed
Demographic module				
Critical threshold of carbon reserves at the end of the year for reproduction	*sB*_res_	100	gC m_soil_^−2^	Fixed
Critical threshold of carbon reserves at the end of the year	CumCR	45	gC m_soil_^−2^	Fixed
Maximal difference between carbon needs and carbon reserves before budburst	bbCR	160	gC m_soil_^−2^	Fixed
Rate of seed production	*R*_SP_	0.05	–	Fixed
Cost to produce one seed	C	0.45	gC	Han et al. (2011)
Rate of empty seeds	*r*_ES_	0.33	–	Oddou-Muratorio (personal observations)
Rate of seed germination	*r*_SG_	0.485	–	Oddou-Muratorio (personal observations)
Rate of seed survival	*r*_SS_	0.15	–	
Average distance of seed dispersal	Δ*s*	18.13	m	Bontemps et al. (2013)
Shape of the seed dispersal kernel	*b*_S_	0.31		Bontemps et al. (2013)
Average distance of pollen dispersal	*δ*_p_	37.9	m	Bontemps et al. (2013)
Shape of the pollen dispersal kernel	*b*_P_	0.97		Bontemps et al. (2013)
Parameter relating tree diameter and male fertility	*γ*_m_	0.82	–	Bontemps et al. (2013)
Rate of selfing	*S*	0.025		Bontemps et al. (2013)
Mean height of newly recruited tree	μ_H_	9	m	P. Dreyfus (personal communications)
Standard deviation of newly recruited tree height	SD_H_	0.34	m	P. Dreyfus (personal communications)
Mean diameter at breast height (DBH) of newly recruited tree	μ_DBH_	13.8	cm	P. Dreyfus (personal communications)
Standard deviation of newly recruited tree DBH	SD_DBH_	0.9	cm	P. Dreyfus (personal communications)

#### The demographic module

##### Adult growth, mortality, and seed production

The reserves produced by photosynthesis at a daily time step were allocated to growth and used to predict tree mortality. Two levels of carbon reserves were considered: the carbon reserve at the end of the year (CumCR; Table 1) and the difference between the carbon reserves before budburst and the amount of carbon required for the complete development of new leaves (bbCR; Table 1). Below a critical level of one of these two indicators, a tree would die. Critical levels of CumCR and bbCR were estimated based on mortality rates assessed on Mount Ventoux (H. Davi unpublished results).

Biomass allocated to wood between the date of budburst and leaf senescence was converted into a diameter at breast height (DBH) increment. Finally, at the end of the year, if the biomass of accumulated reserves (*B*_res_) exceeded the critical rate for seed production (*sB*_res_), the reserve was converted into primary seed production (*N*_S_) for each tree as follows:


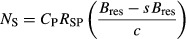
(5)

where *C*_P_ is the crown projection of the tree (Appendix S1), *R*_SP_ is the rate of seed production (Table 1), and *c* is the cost to produce one seed. Parameter *c* was estimated using the dry mass and carbon content of seeds and cupules (Han et al. 2011) assuming an associated respiratory cost of 50%.

The effective seed production of a tree, that is, its female fecundity, was computed as follows:


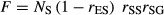
(6)

where *r*_ES_ is the rate of empty seeds, *r*_SS_ is the rate of seed survival, and *r*_SG_ is the rate of seed germination. Parameters *r*_ES_ and *r*_SG_ were calibrated on the basis of a germination experiment in the years 2009–2010, during which 60 seed lots were collected at three elevational levels from Mont Ventoux (20 mother trees per altitude level), and from 100 to 300 seeds per mother tree were scanned (to measure the *r*_ES_) and sown after stratification (to measure the *r*_SG_).

##### Pollen dispersal and mating

Pollen dispersal was modeled by an exponential dispersal kernel describing the probability that a pollen grain emitted at position (0,0) would pollinate a seed tree at distance *r* as follows:


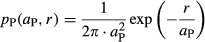
(7)

where the scale parameter *a*_P_ is homogeneous to the mean distance travelled by pollen grain (*δ*_p_) with the relationship *δ*_P_ = 2*a*_P_ (Table 1). The simulation domain was defined with reflecting borders to avoid the loss of border tree progeny.

The contribution *π*_*jk*_ of pollen tree *k* to the outcrossing pollen cloud to the fertilization of seed tree *j* (*j*≠*k*) depended on the distance between trees *j* and *k* through the pollen dispersal kernel *p*_P_ and on its pollen fecundity as related to the diameter DBH_*k*_ as follows:


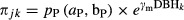
(8)

where *γ*_m_ is related to the diameter effects on male fertility. We assumed no pollen limitation.

In addition to outcrossing, selfing was considered to occur at a fixed rate *s*. Tree *j* was self-pollinated with probability *s* and pollinated by other individuals with probability (1−s) *π*_*jk*_ (for 1 ≤ *k*≤*N* and *k*≠*j*), where *N* is the total adult population size. Parameters *δ*_P_, *s*, and *γ*_m_ were estimated for the *F. sylvatica* trees on Mont Ventoux (Oddou-Muratorio et al. 2010).

##### Seed dispersal and recruitment (density-dependent mortality)

The simulation domain was divided in squared cells to model seed dispersal. The intensity of seed rain from a given seed tree *j* on the center of cell *i* was expressed by


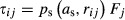
(9)

where *p*_S_ is the seed dispersal kernel, *r*_*ij*_ is the distance from tree *j* to the center of cell *i*, and *F*_*j*_ is the female fecundity of seed tree *j*. The seed dispersal kernel was modeled as pollen dispersal using the exponential kernel described by eqn 4. From *τ*_*ij*_, we computed the number of new trees *N*_*ij*_ from a given seed tree *j* on the whole cell *i* of area *S*, as detailed in Appendix S2.

Within each cell *i*, Σ*jN*_*ij*_ individuals (*j *=* *1 to the total number of seed trees) were created at the age of 40 years, thus assuming that the phenology selection did not proceed differently before and after this life stage. The height and diameter of newly created trees were drawn in a Gaussian distribution of parameter {μ_H_; SD_H_} and {μ_DBH;_ SD_DBH_}, respectively. The spatial position of each new tree was allocated randomly within the cell unless its mother tree was in the same cell *i*; in this case, spatial positions were drawn in a Gaussian dispersal kernel around the position of the mother tree. Mortality during recruitment was modeled as a spatial, random (i.e., independent from TBB), density-dependent process considering that no tree pairs could have more than 30% overlapping crown.

#### A quantitative genetic module for the TBB

The variation in TBB between individuals depended on both (i) the individual genetic variation in *F*_critBB_ and (ii) environmental variation among individual locations and years. In a given environment, the higher is the *F*_critBB_, the later is the TBB. In most of the simulated scenarios, the environmental component of TBB variation was fully determined by the variation in daily temperatures during the spring, which varied across years for a given individual, and across elevations for different individuals. Note that in our simulations, elevation could vary by 200 m between individuals within the same population (see paragraph ‘Simulation result analyses’ below), and thus, both the variations in *F*_critBB_ and elevation contribute to the variation in TBB within population.

The value of *F*_critBB_ was determined by ten independent biallelic loci with purely additive effects. The occurrences of mutations and new allele immigration from other populations than those simulated were ignored. The contribution of a genotype at a given locus l to *F*_critBB_ was given as *m*_l_+*α*_l_*, m*_l_, and *m*_l_*−α*_l_ for the homozygote *A1A1,* the heterozygote *A1A2*, and the homozygote *A2A2,* respectively. All the *m*_l_^*’*^s were identical and equal to 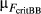/10 (Table S1). We followed the method proposed by Bost et al. (2001) to generate the distribution of allelic effects. They showed that for *N*_l_ loci having an equal *m*_*l*_ = μ/*N*_l_ contribution to the trait, an L-distribution of QTL effects could be simulated with allele effects randomly drawn from a Gaussian distribution of mean μ/2*N*_l_ and with a standard deviation *σ* small enough to ensure that *α*_l_ belongs to [0; μ l^−1^] (here, *σ *= μ/8*N*_l_). Allelic effects *α*_l_ were constant over time (Table 1). The genetic variance for *F*_critBB_ within the population/subpopulation was computed as follows: 
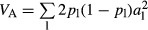
, where *p*_l_ is the frequency of allele A1 at locus l.

In most scenarios with microevolution, we considered *F*_critBB_ to be fully heritable; the narrow-sense heritability 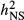 of *F*_critBB_ was set to 1, meaning that the phenotypic variance of *F*_critBB_ (*V*_P_) equaled to the additive genetic variance *V*_A_ (as 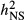 = *V*_A_/*V*_P_), and that the environmental variance *V*_E_ was 0. Each individual *F*_critBB_ value was the sum of genotypic contributions (see above) across the 10 loci. In the control scenario, *F*_critBB_ was variable but not heritable (i.e., *V*_A_ = 0); to match the phenotypic variation obtained in microevolution scenarios, *V*_P_ was set to *V*_E_ = 22. Individual *F*_critBB_ values were randomly drawn from a Gaussian distribution of mean 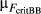 and of variance *V*_P_. Finally, we also considered the case where *F*_critBB_ was itself a quantitative trait with 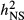 = 0.6 (Kramer et al. 2008); each individual *F*_critBB_ value was the sum of an additive genetic component and of a stochastic environmental component, drawn in a Gaussian distribution of mean 0 and of variance *V*_E_, so that *V*_E_ = (1 − 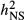) *V*_P_ and *V*_A_ = 22.

### Simulation design and testing hypothesis

**Figure 2:**
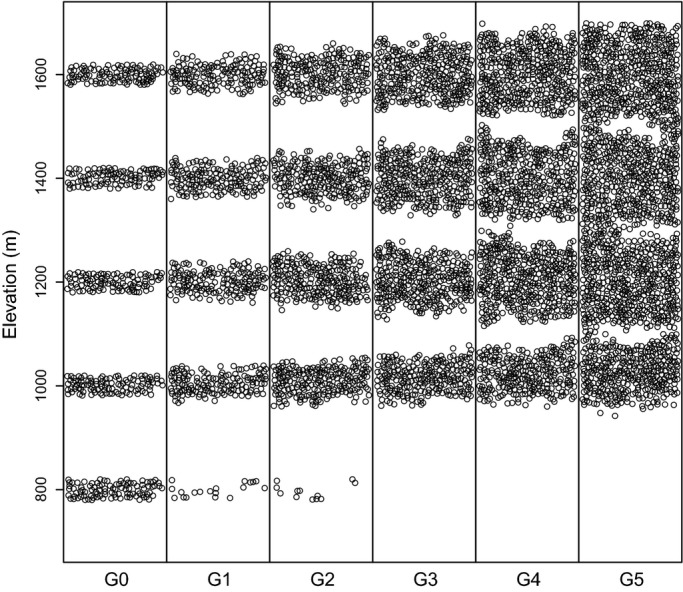
Spatial population dynamics over five generations (from G0 to G5) under ‘adaptive evolution’ scenario (B) along the elevational gradient.

We applied PDG to simulate the evolutionary dynamic of *F. sylvatica* along an elevational gradient from 700 to 1700 m on a 20 ha grid (200 × 1000 m) divided into 500 cells (20 × 20 m) (Fig. 2). This case study mimic an elevational gradient located on Mont Ventoux (southeastern France, 44°10′28′’N; 5°16′16″E), where *F. sylvatica* recently expanded under the black pines (*Pinus nigra*) that were planted at the end of the nineteenth century. The species currently extend from 750 to 1700 m in elevation on the northern aspect. Environmental, climatic and ecological data were available from previous studies (Cailleret and Davi 2011; Davi et al. 2011).

#### Initial conditions and neutral pre-evolution

The initial population included 500 trees (all 40 years old). Their initial height and diameter were drawn by following a Gaussian distribution with parameters {μ_H_; SD_H_} and {μ_DBH_; SD_DBH_} (Table 1). Initial allele frequencies were drawn from a uniform distribution [0,1], and they formed genotypes according to the Hardy–Weinberg equilibrium. The initial population was split into five equal subpopulations at the following five elevational ranges: 790–810, 990–1010, 1190–1210, 1390–1410, and 1590–1610 m (Fig. 2). To introduce initial genetic differentiation among subpopulations and spatial genetic structure within subpopulations similar to the levels observed on the field, we first simulated five generations without selection in which the allelic frequencies within and among subpopulations evolved only as a result of genetic drift and gene flow (Appendix S3).

#### Adaptive evolution over six generations

After initialization, we simulated six nonoverlapping generations (from G0 to G5) of evolution with selection in addition to genetic drift and gene flow. Selection occurred within each generation both through differential mortality and differential reproduction of individual trees. Each generation lasted 70 years and included (i) a seedling stage (from years/ages 0 to 39) and (ii) an adult stage (from years/ages 40 to 70). Adult trees grew for 20 years without reproducing (i.e., until age 60) and then grew and reproduced over the course of 10 years. After 70 years, all the surviving trees were removed. Seeds produced during generation *G*_*x*_ were sent into dormancy until the beginning of generation *G*_x+1_, when survival depended on the total seedling density.

#### Climatic data

The basis climate variables (*X*_basis_) were assessed from 2002 to 2006 using daily meteorological data measured at a permanent weather station located in Ventoux (Porté et al. 2004). The elevation effects on temperature, relative humidity and precipitation were estimated using linear models and data acquired from April 2007 to October 2009 using five HOBO Pro V2 microloggers, which were located at 995, 1117, 1225, 1340, and 1485 m on the north face (Cailleret and Davi 2011), as follows (Table S2):


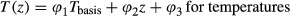
(10)


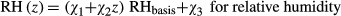
(11)


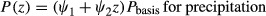
(12)

This 5-year climate sequence (from 2002 to 2006) was repeated in loops (six loops) for 30 years at each generation.

#### Testing of hypotheses

This study investigated how phenotypic plasticity and genetic adaptation contributed to TBB variation across elevations by comparing the following scenarios:

Scenario *A* (‘*Neutral*’) was the baseline scenario without adaptive evolution, because *F*_critBB_ was variable but not heritable (*h*^2^ = 0). Selection occurred within a generation but was not expected to result in *F*_critBB_ changes between generations.
Scenario *B* (‘*Adaptive evolution*’), in which *F*_critBB_ was variable and heritable (*h*^2^ = 1) and selection occurred, potentially resulting in genetic evolution across generations.
Several variants of scenario B were also considered as follows:
Scenario *C* (‘*Evolution without mortality*’), in which *F*_critBB_ was variable and heritable (*h*^2^ = 1) and selection occurred only through differential reproduction without mortality.
Scenario *D* (‘*Evolution without differential reproduction*’), in which *F*_critBB_ was variable and heritable (*h*^2^ = 1) and selection occurred only through differential mortality without differential reproduction among individuals.
Scenario *E* (‘*Evolution, mortality driven by low level of cumulated carbon reserve*’), in which mortality only occurred when the carbon reserve at the end of the year (CumCR) fell below a critical value (Type I mortality, Table 1).
Scenario *F* (‘*Evolution, mortality driven by low level of carbon reserve at budburst*’), in which mortality only occurred when the carbon reserve before budburst (bbCR) fell below a critical value (Type II mortality, Table 1).
Scenario *G* (‘*Evolution, reduced heritability*’), in which the heritability of *F*_critBB_ was set to *h*^2^ = 0.6.
Scenario *H* (‘*Evolution with frost effect on LAI*’), in which every late frost reduced the LAI (Ha) by 10% or (Hb) by 20% per degree below the critical minimal temperature.

For each scenario, we ran 21 repetitions with different random initial conditions. Among the repetitions, only the spatial locations and the 500 initial founder genotypes changed, whereas the allelic effects at each QTL were the same (Table 1). The average genetic value for *F*_critBB_ in the initial founder population was always 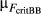 = 190. The same allelic effects were used for all replicate runs of the simulation.

### Simulation result analyses

The continuous population ranging between 700 and 1700 m in elevation was split into five discrete adjacent populations, namely Alt1 (700–900 m), Alt2 (900–1100 m), Alt3 (1100–1300 m), Alt4 (1300–1500 m), and Alt 5 (1500–1700 m). Output variable distributions were obtained for each tree from the 30-year sequence (from ages 40 to 70).

We computed the change in *F*_critBB_ and TBB between generations G0 and G5 (Cb) population per population. As *F*_critBB_ was constant across the lifetime for a given tree, the change in *F*_critBB_ was estimated as follows:


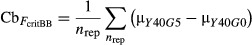
(13)

where *n*_rep_ is the number of repetitions (here *n*_rep_ = 5), and μ_*YnGx*_ is the average *F*_critBB_ value at year *n* of generation X within the population under consideration (*Y*40 corresponded to the first year of the adult life stage).

By contrast, TBB varied among the different climatic years (2002–2006). The change in TBB was estimated as follows:


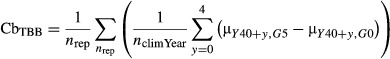
(14)

where *n*_climYear_ = 5, and *y* = 0 for year 2002 up to *y* = 4 for year 2006.

To measure the strength of within-generation selection, we also computed the change within each generation *Gx* as follows for *F*_critBB_:


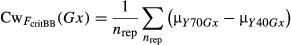
(15)

Note that both Cw and Cb are classically used in quantitative genetics; Cw is also referred to as the selection differential (the difference of the mean trait value in a population before and after an episode of selection), and Cb measures the response to selection (the difference between the population distribution before selection and the distribution of the trait in the next generation). Cw and Cb values were compared between populations and scenarios using simple linear models with interaction between populations and scenarios (Appendix S4). All analyses were performed with R (RDevelopmentCoreTeam 2010).

## Results

Because population Alt1 collapsed in almost all simulations (because of strong mortality below 800 m), it was excluded from the population-level results.

### Plastic response to the climatic gradient (scenario A)

**Figure 3:**
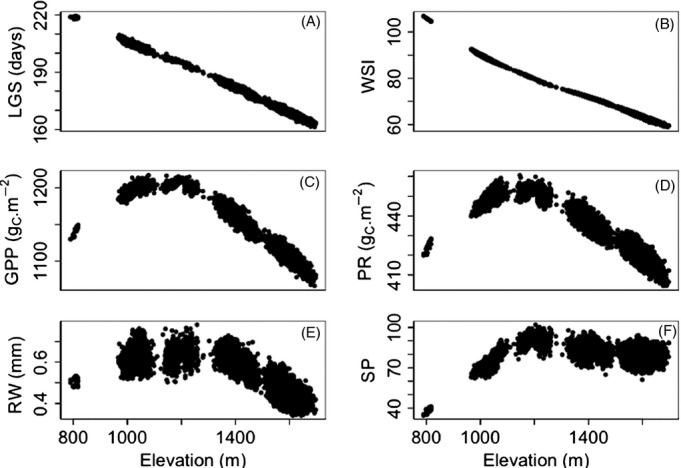
Elevational plastic variation in (A) length of growing season (LGS), (B) water stress index (WSI), (C) gross primary production (GPP), (D) plant respiration (PR), (E) ring width (RW), and (F) seed production (SP). Each point corresponds to the average value across climatic years of the variable of interest for surviving trees (scenario B).

Only surviving trees were used for this analysis. Across all climatic years and all adult life stages, the length of growing season decreased on average from 210 to 160 days (Fig. 3A), and the WSI decreased by 33% between 1000 and 1700 m (Fig. 3B). As a consequence of these two limiting factors, the highest photosynthesis level (GPP = 1216 g_C_ m^−2^) occurred at 1078 m (Fig. 3C) and was almost as high from ∼1050 to 1300 m. However, as respiratory costs also strongly decreased above 1200 m (Fig. 3D), the highest ring increment and seed production values were found at 1258 m (Fig. 3E) and 1204 m (Fig. 3F), respectively. At elevations >1400 m, the ring width decrease was steeper than the seed production decrease. The minimal value of carbon reserves at the end of the year occurred between 1160 and 1420 m, and the greatest difference between carbon reserves and carbon demand during budburst was found below 1000 m (Fig. S1). Mortality was higher, on average, at low/intermediate elevations (Table S3).

The physiological response to the elevational gradient varied significantly among climatic years. For instance, ring widths were large regardless of elevation in 2002, but in 2003, they increased continuously with the elevation, and in 2004, 2005, and 2006, they reached a maximum at 1000, 1100, and 1200 m, respectively (Fig. S2A). This variability among years was also observed for seed production and the level of carbon reserves at the end of the year (Fig. S2B).

### Adaptive response of *F*_critBB_ to the climatic gradient

**Figure 4:**
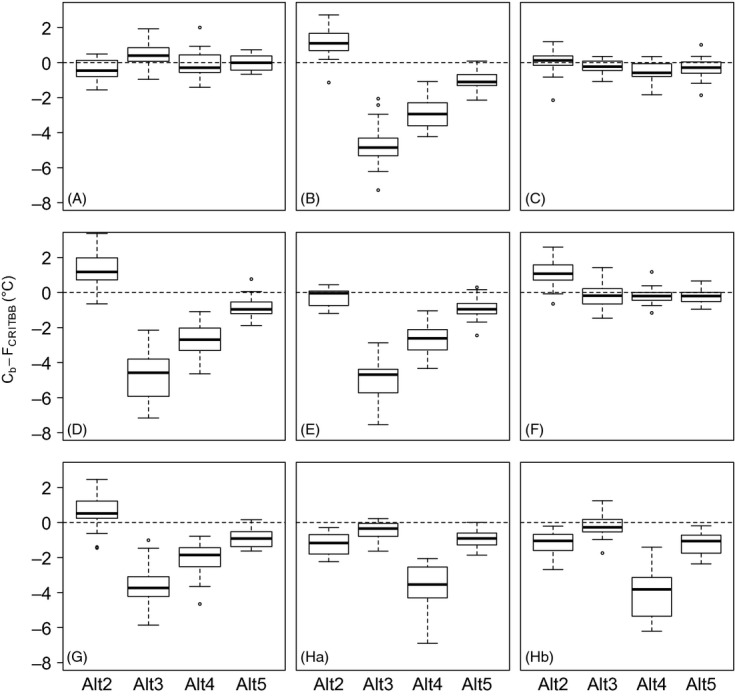
Change in the temperature sum required for budburst (Cb−*F*_critBB_) between generations G0 and G5 within populations Alt2 to Alt5 for different scenarios (letter above each graph). The boxplot represents variation across the 21 repetitions. The dashed line correspond to Cb = 0 (no change). Population Alt1 was removed because of the low number of surviving individuals at G5.

To investigate the effect of genetic adaptation along the gradient, we compared scenario B (adaptive evolution with 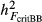 = 1) with scenario A (neutral with 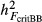 = 0). First, we observed significant differences among scenarios and populations for the patterns of changes in *F*_critBB_ between generations G0 and G5 (denoted 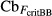), which measures the response to selection (Table 2, Fig. 4A,B, Appendix S4). In scenario A, 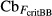 was low (<1°C) in all populations. In scenario B, absolute 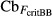 values were significantly higher than in scenario A in populations Alt2 to Alt 4 (Appendix S4). Populations Alt3 and Alt4 evolved toward lower *F*_critBB_ values (on average, 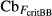 *=* −4.72°C in Alt3 and −2.85°C in Alt4, Fig. 4), while population Alt2 evolved toward a higher *F*_critBB_ (on average, 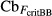 *=* +1.18°C).

**Table 2:** Simulated patterns of evolution for the temperature sum required for budburst (*F*_critBB_). For each population, the changes in *F*_critBB_ within generation G0 (Cw) measures the intensity of selection, while the change between generations G0 and G5 (Cb) measures the response to selection. The phenotypic variance for *F*_critBB_ (*V*_P_) was computed at the last year of generation G5; in scenarios with *h*^2^ = 1 (B to F; Ha and Hb), *V*_P_ is also the additive variance *V*_A_. In scenario A, *V*_A_ = 0; in scenario *G*, *V*_P_ = *V*_A_ + *V*_E_. Population Alt1 is not shown because of low population size.

	Alt2			Alt3			Alt4			Alt5		
Population <	Cb	Cw	*V*_P_	Cb	Cw	*V*_P_	Cb	Cw	*V*_P_	Cb	Cw	*V*_P_
A – Neutral	−0.42	1.32	21.68	0.48	−4.28	18.11	−0.08	−1.06	21.13	0.00	−0.15	21.37
B – Adaptive evolution	1.18	1.57	21.21	−4.72	−3.61	13.60	−2.85	−1.11	20.18	−1.06	−0.21	19.64
C – Evolution without mortality	0.00	0.00	21.15	−0.24	0.00	19.43	−0.52	0.00	20.51	−0.31	0.00	21.10
D – Evolution without differential reproduction	1.33	1.36	21.68	−4.76	−3.47	14.55	−2.69	−0.81	19.40	−0.88	−0.15	20.41
E – Evolution, Type I mortality	−0.23	0.00	19.64	−4.93	−3.72	14.81	−2.63	−1.06	19.03	−0.94	−0.18	20.39
F – Evolution, Type II mortality	0.55	0.98	20.34	−0.03	−0.01	19.81	0.03	0.00	20.50	0.06	0.00	19.98
G – Evolution, reduced heritability	0.60	1.88	33.72	−3.79	−6.09	24.84	−2.07	−1.60	32.31	−0.90	−0.35	31.49
Ha – Evolution, moderate effect of frost	−1.21	−0.40	18.37	−0.41	−0.02	20.30	−3.71	−2.07	17.43	−0.93	−0.30	18.37
Hb – Evolution, strong effect of frost	−1.15	−0.39	18.61	−0.21	0.03	19.42	−4.01	−3.15	18.21	−1.17	−0.33	19.57

**Figure 5:**
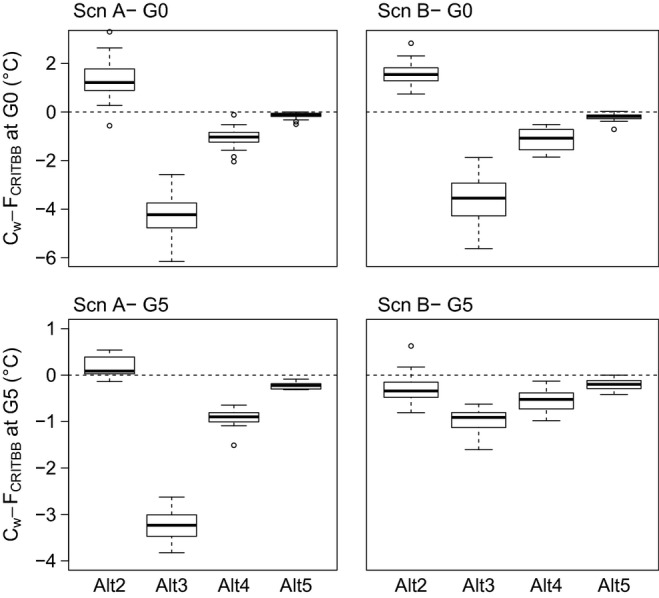
Change in the temperature sum required for budburst (*F*_critBB_) within generation G0 (top panels) and G5 (bottom panels), for scenarios A (neutral, left panels) and B (adaptive evolution, right panels).

Secondly, we investigated patterns of change in *F*_critBB_ within each generation (Cw), as a measure of the strength of selection (Table 2, Fig. 5). Patterns of 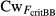 were similar among scenarios A and B at generation G0, with negative 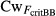 values within populations Alt3 to Alt5 (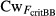 = −3.61°C, in Alt3, scenario B), and a positive 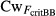 value in population Alt2 (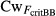 = +1.57°C, scenario B). Differences in absolute 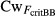 values indicate that selection was twofold more intense in population Alt3 than Alt2 or Alt4, and it was weak in population Alt5. These variations in selection direction and strength were consistent with the variations in *F*_critBB_ observed among generations (Cb) in scenario B. Finally, patterns of 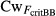 at generation G5 differed among scenarios A and B. While 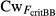 values remained similar across generation in scenario A, 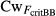 values decreased across generations in scenario B as a consequence of selection and recombination (Fig. 5). The phenotypic variances for *F*_critBB_ within each population were also slightly lower in scenario B than in scenario A at generation G5, in particular in Alt3 (Table 2).

### Deciphering the mechanisms driving microevolution of *F*_critBB_

We investigated the effects of differential reproduction and differential mortality on evolutionary dynamics by comparing scenarios A, B, C, and D. Scenario without mortality (scenario C) led to weak changes in *F*_critBB_ from G0 to G5 (Fig. 4C), but overall the pattern of 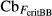 did not significantly differ from scenario A (Appendix S4), indicating that the absence of mortality prevented genetic adaptation. In contrast, scenario without differential reproduction (scenario D) resulted in a change in *F*_critBB_ from G0 to G5 that was as important as it was in scenario B (Fig. 4D), indicating that differential reproduction between trees played a minor role in simulated patterns of adaptation.

We also investigated the variations in 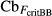 in scenario E, where mortality was driven by low levels of accumulated carbon reserves (Type I mortality), and scenario F, where mortality was driven by low carbon reserve levels at budburst (Type II mortality). In scenario E, populations Alt3 and Alt4 still evolved significantly toward a lower *F*_critBB_ as compared to scenario A, but the population Alt2 did not evolve toward higher *F*_critBB_ (Fig. 4E, Appendix S4). This trend was caused by the absence of Type I mortality at low elevations (Table S3). In scenario F, population Alt2 still evolved toward a higher *F*_critBB_, but populations Alt3 and Alt4 did not evolve toward a lower *F*_critBB_ (Fig. 4F). This result was due to the absence of Type II mortality in these populations (Table S3). The patterns of 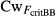 values within populations in scenarios F and G were consistent with these Cb patterns (Fig. S3).

Mortality not only drove evolution in *F*_critBB_ but also affected the elevational range of the population. In scenarios A and D, the range of the whole population (as measured by the average elevation) shifted by on average +202 and +168 m, respectively, between generations G0 and G5 (with minimal elevation ∼800 m), which could be due to higher mortality/lower reproduction at low elevation. By contrast, in scenarios C and E, the elevational shift of the whole population was reduced (+37 and +16 m respectively), and the minimal elevation at G5 was ∼720 m.

### Effect of heritability on genetic adaptation

The heritability-level effects of *F*_critBB_ on the microevolutionary patterns of *F*_critBB_ were analyzed by comparing scenario B (*h*^2^ = 1) to scenario G (*h*^2^ = 0.6). The divergence among populations that evolved positive (Alt2) versus negative (Alt3 and Alt4) values for *F*_critBB_ was reduced when heritability was lower (Fig. 4). However, the response to selection remained important in populations Alt3 and Alt4 (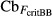 = −3.79°C and −2.07°C, respectively).

### Impact of frost on evolutionary dynamics

We investigated the effects of frost using scenarios including a moderate (scenario Ha) or strong frost effect (scenario Hb) on the LAI. The patterns of microevolution markedly changed when compared to scenario B (Fig. 4). Population Alt2 no longer evolved toward higher values for *F*_critBB_, and population 3 no longer evolved toward lower values of *F*_critBB_. Only population Alt4 evolved toward a lower *F*_critBB_ value (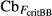 = −4.96°C and −3.55°C for scenarios Ha and Hb, respectively). The Cw patterns for *F*_critBB_ also consistently indicated that selection was strongest in population Alt4 (Fig. S3). Finally, frost effect promoted a reduced elevational shift of the whole population for scenarios Ha and Hb (+75.1 and +27.1 m, respectively) in comparison with scenario B (+167 m), which resulted from less severe mortality at lower elevations (Table S3).

## Discussion

### Effects of plasticity and microevolution on TBB variation

Trees are long-lived species, and they experience a high variability in environmental conditions during their lives because of differences between the juvenile and adult stages and variations among climatic years. Accordingly, trees are expected to display a high plasticity for a wide range of functional traits, including TBB. In our baseline neutral scenario (A), the TBB varied by 12.2 days, on average, between the two extreme climatic years (and therefore 7.6 days degree^−1^) and by 35.2 days between the two extreme elevations (and therefore 5.4 days degree^−1^). This plastic variation was within the TBB range reported for *F. sylvatica* by Vitasse et al. (2011), who found a range of TBB from 4.9 to 5.8 days degree^−1^ across an elevational gradient from 131 and 1533 m in the Pyrenees. However, a lower plasticity was found in previous studies (between 2 and 2.5 days degree^−1^; Kramer et al. 2008; Menzel et al. 2001).

In comparison with the plasticity, the TBB variation resulting from microevolution was small. Between the two most genetically differentiated populations (e.g., between populations Alt2 and Alt3 at generation G5) in the baseline scenario of adaptive evolution (B), the difference of 5.9°C in the temperature sum required for budburst (F_critBB_, the genetic component of TBB) corresponded to an average of 2 days for TBB (Fig. S4). These small variations are consistent with previous experimental studies; Vitasse et al. (2009b) found that a phenological model with constant parameters is able to reproduce TBB for different populations, suggesting that plasticity hides local adaptation. Using a common garden experiment, Vitasse et al. (2009b) also reported that the genetic difference in TBB between populations originating from different elevations was almost 4 days, which is above our estimation of 2 days due to a wider elevational range. However, it is also likely that more differentiation would be found when simulating responses over more than five generations.

### Nonmonotonic elevation effects on the TBB genetic optimum

Although they were weaker than phenotypic differentiation, significant patterns of genetic differentiation for TBB across elevations were nonetheless obtained in only five generations. In scenario B, one population (Alt2) evolved toward delayed budburst (*F*_critBB_ increase of *=* +1.18°C), whereas populations Alt3 and Alt4 evolved toward earlier budburst (*F*_critBB_ decrease of −2.85°C to −4.72°C). The resulting pattern of *F*_critBB_ variation across elevations was thus nonmonotonic. This result occurred because the mortality in population Alt2 was triggered by a lack of sufficient reserves before budburst to produce new leaves (Type II mortality, scenario E), and in populations Alt3 to Alt5, mortality was triggered by low reserves during the winter (Type I mortality, scenario F). The variability across elevations in mortality-triggering factors resulted from the nonlinear variability of the underlying ecophysiological processes, which makes the trees located at different elevations pass different thresholds to mortality during different years. Type I mortality occurred mainly during 2004 for trees from populations Alt3 to Alt5, and Type II mortality occurred mainly during 2003 for trees located below 1100 m (i.e., populations Alt2 and Alt1). In population Alt2, fewer trees displayed low reserves during winter in comparison with trees at higher elevations, because of longer growth period. However, these trees also displayed a higher LAI and biomass, which increased the respiratory and construction costs before budburst and made them more vulnerable to Type II mortality.

When frost damage was considered (scenarios Ha and Hb), other nonmonotonic effects were observed. As only trees that initiated budburst could suffer from late frost, frost damage did not occur at the upper elevations (e.g., population Alt4) because delayed budburst protected them from late frosts. The first mechanistic consequence is that frost did not linearly affect the trees across the elevational gradient. Second, reduced LAI from frost damage can either increase or decrease the risk of mortality depending on its absolute effect for the reserve level. The risk of Type II mortality is expected to increase with frost damage because a reduced LAI decreases the carbon assimilation rate. However, a moderate frost in the model can also reduce Type I mortality because reduced LAI can decrease the carbon reserve required during budburst and the water loss during the following summer. This phenomenon explains why, when frost damage was considered, population Alt2 became less sensitive to carbon demand before budburst and did not evolve toward a later budburst as in the baseline scenario B.

This study sheds light on the mechanisms that underlie genetic and phenotypic patterns of TBB variation. Considering the number of underlying mechanisms involved in TBB and their patterns of environmental variation, this study suggests that nonmonotonic genetic patterns of TBB variation should be the rule rather than the exception. Other factors not considered here (for instance, assortative mating induced by variations in reproductive phenology across elevation) should be further studied (Soularue and Kremer 2012).

### Understanding the mechanisms underlying climate adaptation

This study was based on a new mechanistic model coupling physiology, population dynamics, and quantitative genetics to simulate the short-term evolution of functional traits. Because PDG explicitly accounts for climate effects on the water and carbon exchanges of individual trees as a selective pressure, it provides a useful complement to existing evolutionary models of tree population life history traits (Le Corre and Kremer 2003; Kuparinen et al. 2010). More precisely, in PDG as in these other models, individual fitness is the parameter driving the process of adaptation. However, the primary originality of PDG is that individual fitness is an output, which is calculated as the lifetime reproductive success resulting from a combination of functional traits and the environmental context. This was also used (Kramer et al. 2008) to investigate the temporal patterns of microevolution in a single population. We extended this approach here, and we showed how such a mechanistic model can be used to investigate the type and strength of selection mediated by the climate through the estimation of the selection differential (using Cw, the difference in the population before and after an episode of selection).

In PDG, selection occurred both through differential mortality and reproduction of individual trees within each generation. Mortality was found to be the main driver of evolutionary dynamics, with different types of mortality promoting different patterns of adaptation across elevations. Predicting tree mortality is a key and complex issue in tree physiology and ecology because many mechanisms are involved and interact (carbon starvation, cavitation, and pathogens, (McDowell et al. 2008). We chose to model tree mortality according to the carbon starvation hypothesis alone, in which a tree dies when carbon reserves are too low to allow the setup of new leaves in the spring (second threshold, Type II mortality) or to ensure tree functioning during the winter (first threshold, Type I mortality). We excluded two other mechanisms because (i) no major pathogens were observed in our site for *F. sylvatica* and (ii) in Ventoux the minimal hydraulic potential (−2 Mpa) is above the critical pressure causing a 50% loss of conductance (−2.4 Mpa, Herbette et al. 2010).

In contrast, differential reproduction among individuals was found to have a minor role as a driver of evolutionary dynamics. To our knowledge, few models relate reproduction to tree carbon cycles (Génard et al. 2008). Our main hypothesis here was that seed production increases with carbohydrate reserves. This idea is consistent with the higher seed production observed for dominant trees (H. Davi, personal observation) and for years after good climatic years. It is also consistent with the resource supply hypothesis, in which fruit production, especially during mast years, occurs when carbohydrate reserves are sufficient (Yamauchi 1996).

In addition to selection, the outcome of adaptation is also determined by the level of genetic variation available for selection, which depends on the heritability of the trait under selection and on genetic architecture (in particular, the number of QTLs determining the trait's genetic variation). We showed that levels of heritability for the TBB such as those measured for *F. sylvatica* (*h*^2^ = 0.6, Kramer et al. 2008) lead to significant patterns of genetic differentiation for the TBB across elevations. It was out of the scope of this study to investigate genetic architecture effects in detail. Therefore, we chose a simple additive quantitative genetic model with ten independent QTLs for TBB, as in previous studies (Le Corre and Kremer 2003; Kuparinen et al. 2010). However, further investigations into the effects of the quantitative genetic model are needed (Le Corre and Kremer 2012).

### Main shortcomings of the mechanistic Physio-Demo-Genetic model

Admittedly, full PDG evaluation requires the parameterization and evaluation of complex mechanisms of tree physiology, demography, and selection that was beyond the scope of this study. Among the most important shortcomings of this study, we repeatedly reused a short (5-year) climatic period that is clearly unrealistic and potentially biases the estimates of survival between generations. Moreover, this period included specific climatic years, which can have major influence on results. For instance, 2004 was an exceptional drought year that has led to low growth rates of beeches (Cailleret and Davi 2011). Second, PDG does not include competition between adult trees, which is a process that is potentially more important for tree growth and survival than climate. However, not accounting for competition in studying microevolution driven by TBB is reasonable because TBB is related primarily to temperature and only secondarily to competition. Indeed, we previously showed that dominant trees exhibited an earlier TBB, but this effect is small in comparison with the elevation and year effects (Davi et al. 2011). Third, we used nonoverlapping generations, which make individual level comparisons with forest inventory data and tree ring increment measurements difficult. Fourth, dormancy was not taken into account in modeling *F. sylvatica* budburst. Fifth, a real sensitivity analysis on the entire model (as performed for the ecophysiological component of PDG in Dufrêne et al. 2005) will be needed to strengthen some of our conclusions and to draw a more accurate picture of what process control genetic adaptation of budburst.

Nevertheless, valid conclusions could be drawn using the current version of PDG. This success is possible mainly because the physiological module of PDG (CASTANEA), which models the climate effect on tree functioning, has already been thoroughly validated for European beech in several previous studies (e.g., Dufrêne et al. 2005). PDG simulated the maximum tree ring width for elevations between 1100 and 1420 m, which also corresponds to the maximum ring width observed in the field (Cailleret and Davi 2011).

## Conclusions

We described here a new modeling tool (PDG) to assess the potential mechanisms of local adaptation for trees under changing environmental conditions. The primary originalities of the PDG model are that it combines physiology, demography, and genetics and that fitness is a dynamic output of the model. Such complex models are useful tools for predicting the evolution of nonequilibrium forest populations under CC, under which many tipping points and nonlinear effects may be involved. PDG model requires a large amount of data to be parameterized and tested, and results must be cautiously interpreted. Demographic processes such as mortality and reproduction should be further studied, and other processes such as competition and regeneration have to be included in this general framework. Nevertheless, the following two important conclusions emerge from our present study: (i) Genetic evolution of tree populations can occur in a few generations (<5), and (ii) patterns of genetic differentiation across space (and across elevations here) can be nonmonotonic.

## Data archiving statement

The raw simulation data underlying the main results of the study are available from the Dryad Digital Repository: http://dx.doi.org/10.5061/dryad.n80f4.

## Supporting Information

**Appendix S1.** Supplement equations required for the individualbased version of CASTANEA model. **Appendix S2.** Detailed equations for the number of seedlings from seed tree *j* dispersed on cell *i (N_ij_)*. **Appendix S3.** Genetic differentiation among subpopulations and genetic structure within subpopulation after pre-evolution. **Appendix S4.** Statistical model for the comparison of simulated patterns of evolution among populations and scenarios.

**Table S1.** Parameters for allelic effects in the quantitative genetic module. **Table S2.** Parameters related to the effects of elevation on the local climate. **Table S3.** Demographic dynamics for populations Alt1 to Alt5 under the different scenarios. **Figure S1.** Simulated minimal reserve levels at the end of the year (A) and maximum differences between carbon demand and carbon reserves before budburst (B) across elevations. **Figure S2.** (A) Average ring width and seed production by population for different climatic years. (B) Average reserve at the end of the year and difference between carbon demand and reserves before budburst by population for different climatic years. **Figure S3.** Change within generation G0 of *F*_critBB_ (Cw_FcritB_) within each population for Alt2 to Alt5 different scenarios (letter above each graph). **Figure S4.** Change in TBB from generations G0 to G5 (Cb_TBB_) within each population Alt2 to Alt5 for different scenarios (letter above each graph).
