# Supplementary material for: Simulating local adaptation to climate of forest trees with a Physio-Demo-Genetics model
Source: Evol Appl. 2014 Feb 21;7(4):453–67. doi: 10.1111/eva.12143 (PMC4001444; doi:10.1111/eva.12143)
Supplement: Supplementary file 1 — Appendix S1. Supplement equations required for the individualbased version of CASTANEA model. Appendix S2. Detailed equations for the number of seedlings from seed tree j dispersed on cell i (Nij). Appendix S3. Genetic differentiation among subpopulations and genetic structure within subpopulation after pre-evolution. Appendix S4. Statistical model for the comparison of simulated patterns of evolution among populations and scenarios. [file eva0007-0453-sd1.docx]

# Online Appendix 1: Supplement equations required for the individual-based version of CASTANEA model

Initial individual tree biomass (*B_tree_*) was deduced from tree volume (*V_tree_*) and average wood density (ρ_wood_) as:

The tree volume (*V_tree_*) was computed as the sum of the volume of the trunk (*V_trunk_*) and of branches and small wood (*V_branches_*). *V_trunk_* was first estimated from diameter at breast height (*DBH_tree_* in cm) and tree height (*H_tree_* in XX) using the empirical equation:

$\text{V}_{\text{trunk}}=\left(\text{α}_{1}\text{DBH}_{\text{tree}}^{2}\text{H}_{\text{tree}}+\text{α}_{2}\text{H}_{\text{tree}}\text{DBH}_{\text{tree}}^{3}+\text{α}_{3}\text{DBH}_{\text{tree}}^{2}\text{H}_{\text{tree}}^{2}\right)\left(1+\left(\frac{\text{α}_{4}}{\text{DBH}_{\text{tree}}^{3}}\right)+\text{α}_{5}\text{DBH}_{\text{tree}}^{2}+\frac{\text{α}_{6}}{\text{H}_{\text{tree}}}+\text{α}_{7}\text{H}_{\text{tree}}\right)$where the α’s are parameters given in Table A1.1

*V_branches_* was then derived from V_trunk_ as follows :

$$V_{branches}=\beta V_{trunk}$$

Where β is a parameter depending on tree circumference (Table A1.1)

After one year of growth new circumference at breast height (C130) and height (H_tree_) of each tree were estimated as follows:

$\log\left(C130\right)=\sigma_{1}\log\left(B_{tree}\right)+\sigma_{2}$ and $H_{tree}=\sigma_{3}\log\left(C130\right)+\sigma_{4}$

Crown projection (C_P_) was assessed from diameter at breast height using unpublished data from previous measurements for *Fagus sylvatica* in Fontainebleau, France (see (Davi *et al.*, 2008) for a description of the site).

$$C_{p}=\mu_{1}DBH_{tree}+\mu{}_{2}$$

The maximum of leaf area developed during a year (LAI_tree_) was assessed for each tree from carbon reserves (Davi *et al.*, 2009) and basal area at breast height for a tree (G_tree_) as:

$$LAI_{tree}=\lambda_{1}+\lambda_{2}G_{tree}^{\lambda_{3}}\left(\frac{B_{res}}{B_{th res}}\right)^{\lambda_{4}}$$

This equation differs from the logistic one used in Davi et al. (2009), because here LAI is computed at tree scale and not at stand level.

Basal area at breast height of a given tree (G_tree_) was estimated using crown projection and its own diameter (in cm).

$$G_{tree}=1000\frac{pi}{C_{p}}\left(\frac{DBH_{tree}}{200}\right)^{2}$$

**Table A1.1:** Parameters used to compute biomass using empirical equations

| **Parameters of relationships between trunk volume and tree diameter and tree height** | |
| --- | --- |
| α_1_ | 3.999957e-5 |
| α_2_ | 1.09819e-7 |
| α_3_ | -2.82354e-7 |
| α_4_ | -2.41275e4 |
| α_5_ | 1.37238e-5 |
| α_6_ | 1.04979 |
| α_7_ | 2.85037e-3 |
| **Parameters of relationships between trunk and branches volumes** | |
| Range of tree circumference (mm) | β Values |
| [0, 31] | 0.316 |
| ]31, 47] | 0.05 |
| ]47, 63] | 0.062 |
| ]63, 79] | 0.09 |
| ]79, 94] | 0.104 |
| ]94, 110] | 0.11 |
| ]110, 126] | 0.106 |
| ]126, 141] | 0.1 |
| ]141, 157] | 0.092 |
| ]157, 173] | 0.087 |
| ]173, 188] | 0.082 |
| ]188, 204] | 0.081 |
| >204 | 0.08 |
| **Parameters of relationships between new circumference, height and biomass** | |
| σ_1_ | 0.3888 |
| σ_2_ | 2.142 |
| σ_3_ | 8.1622 |
| σ_4_ | -14.565 |

**Table A1.2:** Parameters required for the individual-based version of CASTANEA model

| **Acronym** | **Variable** | **Unit** | **Value** | **Source** |
| --- | --- | --- | --- | --- |
| ρ_wood_ | Wood density | Kg m^-3^ | 618[550 in the model] | Bontemps (2012)^1^ |
| λ_1_ | Wood area Index |  | 0.5 |  |
| λ_2_ | Allometric effect of G on LAI |  | 1.8 |  |
| λ_3_ | Power effect of G on LAI |  | 0.5 |  |
| λ_4_ | Power effect of carbon reserves on LAI |  | 0.3 |  |
| μ_1_ | Slope of the relationship between crown projection and DBH | m²_leaves ._mm^-2^_wood_ | 1.79 | Davi (2004) |
| μ_2_ | Intercept of the relationship between crown projection and DBH | m² | -5.96 | Davi (2004) |

^1^Average value for 170 trees for rings of the last 30 years from cores obtained at breast height

# Online Appendix 2: Detailed equations for the number of seedlings from seed-tree *j* dispersed on cell *i* (*N_ij_*)

The intensity of the seed rain from a given mother-tree *j* on the center of a cell *i* is given by

$\tau_{ij}=p_{s}\left(a_{s},r_{ij}\right)F_{j}$ (eq. 6, main text)

Where *p*_S_ is the seed dispersal kernel modeled using an exponential (eq. 2), r_ij_ is the distance from tree j to center of cell i, Fj is the female fecundity of mother j.

In a first step we accounted for the fact that the modeled space is finite and that part of the seed rain will be lost in particular for mother-tree close to border (border effect). To reduce border effect we assumed reflecting borders on the four square of the modeled space and correct the intensity of the seed rain from mother-tree *j* on the center of a cell *i* as follows:

$$\tau'_{ij}=\left(p_{s}\left(a_{s},r_{ij}\right)+p_{s}\left(a_{s},r_{Nij}\right)+p_{s}\left(a_{s},r_{Sij}\right)+p_{s}\left(a_{s},r_{Eij}\right)+p_{s}\left(a_{s},r_{Wij}\right)\right)F_{j}$$

Where r_Nij_ r_Sij_ r_Eij_ r_Wij_ are the distances from tree j to center of cell i after reflection respectively on border North, South, East and West.

The second step was to integrate τ’_ij_ to get the seed flux Φ_ij_ from mother-tree *j* on the whole square cell *i* of length *l*. Rather than full integration, Φ_ij_ was estimated as :

Φ_ij =_ *τ’ _ij_×l²*

Then we computed the number of recruited seedlings originating from each mother-tree *j* as:

*N_ij_* = 0 if Φ_ij_ < 1;

*N_ij_*=*RR*(*π’ _ij_*) when Φ_ij_ >1

with *RR* the “random rounding” to the next integer value.

# Online Appendix 3: Genetic differentiation among sub-populations and genetic structure within sub-population after pre-evolution

Rather than reaching a balance between genetic drift and gene flow, the objective of the pre-evolution phase was to introduce initial levels of genetic differentiation among sub-populations and spatial genetic structure within sub-population similar those observed in experimental data sets. This was done by simulating five generations without selection where allelic frequencies within and among subpopulations evolved only due to genetic drift and gene flow. The only process affecting the dynamics was reproduction, following the seed and pollen dispersal kernels described in the main paper, with no variation of male and female fecundity among trees and finite and constant population size of 500 individuals. Detailed analyses below showed that five generations of neutral pre-evolution before G0 were enough to reach these values.

**Genetic differentiation among sub-populations:** To estimate genetic differentiation among subpopulations Alt1 to Alt5, we computed the average pairwise F_ST_ values across the 420 simulated loci (10 QTLs + 400 neutral SNP + 10 neutral microsatellites), and across the 10 QTLs only. The significance of F_ST-_values was assessed using the exact test of population differentiation of (Raymond & Rousset, 1995). All these estimations and tests were performed using Arlequin (Schneider *et al.*, 2000).

After 5 generations (at G0), the average F_ST-_value among populations (Table A3.1) was closed to the one measured in natural population on Mont Ventoux using either neutral microsatellites (F_ST_ = 2.6%, (Lander *et al.*, 2011) or SNPs (F_ST_ = 2.3%, Lalague et al. in prep).

**Table A3.1:**  Pairwise F_ST_ among sub-populations (upper part of the table) and exact p-value for the non-differentiation test (lower part of the Table) at G0 after 5 generations of neutral-pre-evolution. Values averaged over 5 repetitions.

|  | **Alt1** | **Alt2** | **Alt3** | **Alt4** | **Alt5** |
| --- | --- | --- | --- | --- | --- |
| **Alt1** |  | 0.0277 | 0.0270 | 0.0260 | 0.0267 |
| **Alt2** | 1.0000 |  | 0.0264 | 0.0271 | 0.0271 |
| **Alt3** | 1.0000 | 1.0000 |  | 0.0269 | 0.0274 |
| **Alt4** | 1.0000 | 1.0000 | 1.0000 |  | 0.0260 |
| **Alt5** | 1.0000 | 1.0000 | 1.0000 | 1.0000 |  |

**Spatial genetic structure (SGS) within population:** SGS was characterized by analyzing variation of genetic relatedness, as measured by the kinship coefficient (Fij) of Loiselle et al. (Loiselle *et al.*, 1995), as function of the logarithm of geographic distance *d*_ij_ between every pair of adults *i* and *j*. To test SGS, the *F*_ij_ values were regressed on ln(*d*_ij_), where *d*_ij_ is the spatial distance between individuals *i* and *j*, to provide the regression slope *b*. Then, the spatial positions of the individuals were permuted 5,000 times in order to get the frequency distribution of *b* under the null hypothesis that *F*_ij_ and *d*_ij_ were uncorrelated. Following Vekemans and Hardy (2004), the SGS intensity was quantified by *Sp* = *b*/(*F*_1_ – 1), where *F*_1_ is the average kinship coefficient between individuals of the first distance class (< 50 m).

At G0, significant SGS was observed in all repetitions and sub-populations (i.e. b significantly negative). Moreover, the intensity of SGS (as measured by Sp=0.02 on average across sub-populations and repetitions) was similar to what observed in *F. sylvatica* in Mont Ventoux using microsatellites (Sp=0.023, (Oddou-Muratorio *et al.*, 2010)

**Table A3.2:**  Estimates of Spatial Genetic Structure at G0 at G0 after 5 generations of neutral-pre-evolution, as summarized by the slope of the regression of pairwise relatedness on logarithm of distance (b), the average genetic relatedness within the first distance class (Fij_1DC) and the Sp-statistics.

| **Repetition** | **b (se)** | | **Fij_1DC (se)** | | **Sp** |
| --- | --- | --- | --- | --- | --- |
| 1 | -0.0192 | (0.0009) | 0.0431 | (0.0016) | 0.0200 |
| 2 | -0.0167 | (0.0009) | 0.0367 | (0.0016) | 0.0173 |
| 3 | -0.0197 | (0.0009) | 0.0426 | (0.0016) | 0.0206 |
| 4 | -0.0188 | (0.001) | 0.0424 | (0.0016) | 0.0196 |
| 5 | -0.0217 | (0.001) | 0.0462 | (0.0018) | 0.0228 |

# Online Appendix 4: Statistical model for the comparison of simulated patterns of evolution among populations and scenarios.

Simple linear models (lm) were used to analyze the variation of the output variables of interest (e.g. C_b_ and C_w_ for *F_critBB_* /TBB) among scenarios and populations. In this appendix, we detail the model used to investigate statistical differences in the patterns of C_b_-*F_critBB_* between generations G0 and G5 (presented on Fig. 4 on the main document).

**Model for *F_critBB_* at generation G5**

The output variable “C_b_-*F_critBB_*” was modeled as:

C_b_-*F_critBB i,j_=* Intercept *+* Population_i_ + Scenario_j_ + interaction (Population, Scenario)

where 1≤i≤4 (4 populations, Alt2 to Alt5) and 1≤j≤9 (9 scenarios, A to Hb). For all scenarios, 21 estimations of C_b_-*F_critBB_* in each population were available.

We used the R function ‘lm’ to fit this model and estimate the effects using a type III sum of squares. We were mostly interested in testing the significance of the interaction term between population and scenario, as a statistical evidence of divergent trajectories of specific population under a specific scenario. Overall, the three factors “population”, “scenario” and their interaction significantly explained the variation in C_b_-*F_critBB_* (Table A4.1 below). The adjusted mean for each population and each scenario can be obtained by summing the corresponding main effects listed in table A4.2. Because the main effects were estimated taking as reference the Scenario A and the population Alt2, it is possible in some cases to check directly from table A4.2 which adjusted means are significantly different from those produced in scenario A. For instance, the adjusted mean of C_b_-*F_critBB_* in population Alt2 , scenario B is -.42 + 1.6= +1.18 which is significantly higher than the adjusted mean of C_b_-*F_critBB_* in population Alt2 , scenario A.

**Table A4.1:**  ANOVA table of the complete model tested for C_b_-*F_critBB_* at generation G5. The adjusted R² was 0.81.

|  | df | Sum of squares | Mean squares | F-value | p-value |
| --- | --- | --- | --- | --- | --- |
| Population | 3 | 607.11 | 202.37 | 308.51 | <0.001 |
| Scenario | 8 | 500.49 | 62.56 | 95.37 | <0.001 |
| Population* scenario | 24 | 953.16 | 39.71 | 60.55 | <0.001 |
| Residuals | 664 | 448.67 | 0.66 |  |  |

**Table A4.2:**  Detailed effect of each factor on *F_critBB_* at generation G5

| **Factor** | **Main effect** | **t value** | **p-value** |  |
| --- | --- | --- | --- | --- |
| Intercept | -0.423 | -2.335 | 0.020 | * |
| Population Alt3 | 0.902 | 3.521 | <0.001 | *** |
| Population Alt4 | 0.338 | 1.319 | 0.187 |  |
| Population Alt5 | 0.420 | 1.641 | 0.101 |  |
| Scenario B | 1.604 | 6.264 | <0.001 | *** |
| Scenario C | 0.418 | 1.633 | 0.103 |  |
| Scenario D | 1.749 | 6.829 | <0.001 | *** |
| Scenario E | 0.196 | 0.764 | 0.445 |  |
| Scenario F | 0.972 | 3.796 | <0.001 | *** |
| Scenario G | 1.023 | 3.994 | <0.001 | *** |
| Scenario Ha | -0.790 | -3.085 | 0.002 | ** |
| Scenario Hb | -0.728 | -2.843 | 0.005 | ** |
| Alt3×scnB | -6.801 | -18.776 | <0.001 | *** |
| Alt4×scn B | -4.366 | -12.053 | <0.001 | *** |
| Alt5×scn B | -2.658 | -7.340 | <0.001 | *** |
| Alt3×scn C | -1.139 | -3.144 | 0.002 | ** |
| Alt4×scn C | -0.852 | -2.353 | 0.019 | * |
| Alt5×scn C | -0.727 | -2.008 | 0.045 | * |
| Alt3×scn D | -6.993 | -19.306 | <0.001 | *** |
| Alt4×scn D | -4.352 | -12.014 | <0.001 | *** |
| Alt5×scn D | -2.628 | -7.256 | <0.001 | *** |
| Alt3×scn E | -5.607 | -15.479 | <0.001 | *** |
| Alt4×scn E | -2.738 | -7.560 | <0.001 | *** |
| Alt5×scn E | -1.130 | -3.121 | 0.002 | ** |
| Alt3×scn F | -1.481 | -4.089 | 0.000 | *** |
| Alt4×scn F | -0.853 | -2.354 | 0.019 | * |
| Alt5×scn F | -0.913 | -2.520 | 0.012 | * |
| Alt3×scn G | -5.287 | -14.597 | <0.001 | *** |
| Alt4×scn G | -3.010 | -8.310 | <0.001 | *** |
| Alt5×scn G | -1.919 | -5.298 | <0.001 | *** |
| Alt3×scn Ha | -0.095 | -0.263 | 0.793 |  |
| Alt4×scn Ha | -2.836 | -7.829 | <0.001 | *** |
| Alt5×scn Ha | -0.137 | -0.378 | 0.706 |  |
| Alt3×scn Hb | 0.037 | 0.101 | 0.920 |  |
| Alt4×scn Hb | -3.196 | -8.824 | <0.001 | *** |
| Alt5×scn Hb | -0.441 | -1.217 | 0.224 |  |

Signif. codes: <0.001 : ‘***’; <0.01 :‘**’ <0.05 :‘*’; <0.1 :‘.’

The effect of the following terms were set to 0 : Population Alt2,Scenario A, Alt2*A, Alt3*A,Alt4*A, Alt5*A, Alt2*B, Alt2*C, Alt2*D, Alt2*E, Alt2*F, Alt2*G, Alt2*Ha, Alt2*Hb

**References for the online Appendixes**

Davi, H., Barbaroux, C., Dufrêne, E., Francois, C., Montpied, P., Bréda, N., & Badeck, F. (2008) Modelling leaf mass per area in forest canopy as affected by prevailing radiation conditions. *Ecological Modelling*, **211**, 339-349.

Davi, H., Barbaroux, C., Francois, C., & Dufrêne, E. (2009) The fundamental role of reserves and hydraulic constraints in predicting LAI and carbon allocation in forests. *Agricultural and Forest Meteorology*, **149**, 349-361.

Lander, T.A., Oddou-Muratorio, S., Prouillet-Leplat, H., & Klein, E.K. (2011) Reconstruction of a beech population bottleneck history using archival demographic information and Bayesian analysis of genetic data. *Molecular Ecology*, **20**, 5182-5196.

Loiselle, B.A., Sork, V.L., Nason, J., & Graham, C. (1995) Spatial genetic structure of a tropical understory shrub, Psychotria officinalis (Rubiaceae). *American Journal of Botany*, **82**, 1420-1425.

Oddou-Muratorio, S., Bontemps, A., Klein, E.K., Chybicki, I.J., Vendramin, G.G., & Suyama, Y. (2010) Comparison of direct and indirect genetic methods for estimating seed and pollen dispersal in Fagus sylvatica and Fagus crenata. *Forest Ecology and Management*, **259**, 2151–2159.

Raymond, M. & Rousset, F. (1995) GENEPOP: population genetics software for exact tests and ecumenicism. *Journal of Heredity*, **86**, 248-249.

Schneider, S., Roessli, D., & Excoffier, L. (2000) Arlequin ver. 2.000: a software for population genetics data analysis. Genetics and Biometry Laboratory, University of Geneva, Switzerland.

Vekemans, X. & Hardy, O.J. (2004) New insights from fine-scale spatial genetic structure analyses in plant populations. *Molecular Ecology*, **13**, 921-935.
