# Supplementary material for: Simulating local adaptation to climate of forest trees with a Physio-Demo-Genetics model
Source: Evol Appl. 2014 Feb 21;7(4):453–67. doi: 10.1111/eva.12143 (PMC4001444; doi:10.1111/eva.12143)
Supplement: Supplementary file 2 — Table S1. Parameters for allelic effects in the quantitative genetic module. Table S2. Parameters related to the effects of elevation on the local climate. Table S3. Demographic dynamics for populations Alt1 to Alt5 under the different scenarios. Figure S1. Simulated minimal reserve levels at the end of the year (A) and maximum differences between carbon demand and carbon reserves before budburst (B) across elevations. Figure S2. (A) Average ring width and seed production by population for different climatic years. (B) Average reserve at the end of the year and difference between carbon demand and reserves before budburst by population for different climatic years. Figure S3. Change within generation G0 of FcritBB (CwFcritB) within each population for Alt2 to Alt5 different scenarios (letter above each graph). Figure S4. Change in TBB from generations G0 to G5 (CbTBB) within each population Alt2 to Alt5 for different scenarios (letter above each graph). [file eva0007-0453-sd2.docx]

**Simulating local adaptation to climate of forest trees with a Physio-Demo-Genetics model**

Authors: Sylvie Oddou-Muratorio1*, Hendrik Davi1*

**Adresses:** ^1^ INRA, UR629 Ecologie des Forêts Méditerranéennes (URFM), F-84914 Avignon, France

**e-mails:** [oddou@avignon.inra.fr](mailto:oddou@avignon.inra.fr); [hendrik.davi@avignon.inra.fr](mailto:hendrik.davi@avignon.inra.fr)

**Online supplementary tables and figures:**

**Table S1:** Parameters related to the effects of elevation on the local climate

**Table S2:** Parameters for allelic effects in the quantitative genetic module

**Table S3:** Demographic dynamics for populations Alt1 to Alt5 under the different scenarios.

**Figure S1**: Simulated minimal reserve levels at the end of the year (A) and maximum differences between carbon demand and carbon reserves before budburst (B) across elevations. Horizontal red lines indicate the threshold below which mortality occurs.

**Figure S2:** A: Average ring width and seed production by population for different climatic years. B: Average reserve at the end of the year and difference between carbon demand and reserves before budburst by population for different climatic years.

**Figure S3**: Change within generation G0 of *F_critBB_* (Cw*_FcritB_*) within each population for Alt2 to Alt5 different scenarios (letter above each graph).

**Figure S4**: Change in TBB from generation G0 to G5 (Cb_TBB_) within each population Alt2 to Alt5 for different scenarios (letter above each graph).

**Table S1:** Parameters for allelic effects in the quantitative genetic module

| **Parameter** | **Acronym** | **Value** | **Unit** |
| --- | --- | --- | --- |
| Number of loci controlling F_critBB_ | L | 10 | **-** |
| Allelic effect at locus 1 | *α_1_* | 1.377 | °C |
| Allelic effect at locus 2 | *α_2_* | 0.527 | °C |
| Allelic effect at locus 3 | *α_3_* | 0.628 | °C |
| Allelic effect at locus 4 | *α_4_* | 0.736 | °C |
| Allelic effect at locus 5 | *α_5_* | 1.326 | °C |
| Allelic effect at locus 6 | *α_6_* | 1.783 | °C |
| Allelic effect at locus 7 | *α_7_* | 0.138 | °C |
| Allelic effect at locus 8 | *α_8_* | 1.064 | °C |
| Allelic effect at locus 9 | *α_9_* | 2.451 | °C |
| Allelic effect at locus 10 | *α_10_* | 0.716 | °C |

**Table S2:** Parameters related to the effects of elevation on the local climate

| **Climate Variable** | **Parameter** | **Value** |
| --- | --- | --- |
| Average temperature | φ_1_ | 1.02164 |
| Average temperature | φ_2_ | -0.00704 |
| Average temperature | φ_3_ | 7.10665 |
| Minimal temperature | φ_1_ | 1.07896 |
| Minimal temperature | φ_2_ | -0.00649 |
| Minimal temperature | φ_3_ | 7.15669 |
| Maximal temperature | φ_1_ | 0.9969 |
| Maximal temperature | φ_2_ | -0.00756 |
| Maximal temperature | φ3 | 6.98725 |
| Relative humidity | χ_1_ | 0.000123 |
| Relative humidity | χ _2_ | 0.728932 |
| Relative humidity | χ _3_ | 12 |
| Precipitation | ψ_1_ | 0.00096 |
| Precipitation | ψ _2_ | - 0.07445 |

**Table S3:** Demographic dynamics for populations Alt1 to Alt5 under the different scenarios.

The mean elevation of a tree at initiation (z_mean_init_) is the same across scenarios. Final population size (*N_end_*), mean elevation (z_mean_) of a surviving tree in the final population and its standard deviation (sd), average mortality rate over the 6 generations (*M*) were computed as average values over a subset of 21 repetitions.

| **Scenario** | **Code** | **Pop** | z_mean_init_ | ***N_end_*** | **z_mean_ (m)** | **sd** | **M** |
| --- | --- | --- | --- | --- | --- | --- | --- |
| Neutral | A | Alt1 | 800.45 | 1.0 | 816.1 | 6.4 | 0.081 |
|  |  | Alt2 | 1000.84 | 197.4 | 1046.1 | 29.3 | 0.025 |
|  |  | Alt3 | 1200.49 | 115.6 | 1184.6 | 54.3 | 0.054 |
|  |  | Alt4 | 1400.64 | 294.9 | 1420.4 | 49.7 | 0.019 |
|  |  | Alt5 | 1600.54 | 477.9 | 1595.8 | 53.2 | 0.002 |
| Evolution | B | Alt1 | 800.45 | 2.4 | 814.2 | 10.5 | 0.086 |
|  |  | Alt2 | 1000.84 | 201.9 | 1043.8 | 29.9 | 0.024 |
|  |  | Alt3 | 1200.49 | 311.8 | 1194.4 | 55.9 | 0.032 |
|  |  | Alt4 | 1400.64 | 339.9 | 1414.3 | 52.2 | 0.016 |
|  |  | Alt5 | 1600.54 | 481.9 | 1596.9 | 53.2 | 0.002 |
| Evolution  without mortality | C | Alt1 | 800.45 | 341.9 | 805.9 | 37.6 | 0.000 |
|  |  | Alt2 | 1000.84 | 499.7 | 1002.8 | 52.0 | 0.000 |
|  |  | Alt3 | 1200.49 | 546.8 | 1200.7 | 55.5 | 0.000 |
|  |  | Alt4 | 1400.64 | 537.9 | 1400.2 | 55.1 | 0.000 |
|  |  | Alt5 | 1600.54 | 538.0 | 1601.5 | 55.2 | 0.000 |
| Evolution | D | Alt1 | 800.45 | 9.8 | 827.9 | 18.3 | 0.091 |
| without |  | Alt2 | 1000.84 | 209.3 | 1044.9 | 30.8 | 0.025 |
| differential |  | Alt3 | 1200.49 | 268.8 | 1195.1 | 52.9 | 0.037 |
| reproduction |  | Alt4 | 1400.64 | 321.3 | 1413.9 | 50.2 | 0.016 |
|  |  | Alt5 | 1600.54 | 475.7 | 1596.7 | 53.2 | 0.002 |
| Evolution, | E | Alt1 | 800.45 | 322.1 | 804.8 | 37.3 | 0.000 |
| Type I mortality |  | Alt2 | 1000.84 | 472.6 | 1003.2 | 51.8 | 0.000 |
|  |  | Alt3 | 1200.49 | 280.2 | 1192.5 | 54.8 | 0.036 |
|  |  | Alt4 | 1400.64 | 319.9 | 1413.1 | 51.9 | 0.016 |
|  |  | Alt5 | 1600.54 | 458.0 | 1597.7 | 53.1 | 0.002 |
| Evolution, | F | Alt1 | 800.45 | 2.2 | 817.1 | 10.8 | 0.083 |
| Type II |  | Alt2 | 1000.84 | 194.0 | 1044.6 | 30.0 | 0.025 |
| mortality |  | Alt3 | 1200.49 | 513.8 | 1201.3 | 55.5 | 0.000 |
|  |  | Alt4 | 1400.64 | 511.2 | 1400.3 | 55.3 | 0.000 |
|  |  | Alt5 | 1600.54 | 512.7 | 1600.5 | 55.4 | 0.000 |
| Evolution, | G | Alt1 | 800.45 | 2.8 | 819.5 | 16.0 | 0.076 |
| reduced |  | Alt2 | 1000.84 | 195.6 | 1045.4 | 30.5 | 0.025 |
| heritability |  | Alt3 | 1200.49 | 274.0 | 1193.7 | 55.5 | 0.034 |
|  |  | Alt4 | 1400.64 | 315.2 | 1413.2 | 51.8 | 0.017 |
|  |  | Alt5 | 1600.54 | 454.5 | 1596.2 | 53.1 | 0.003 |
| Evolution with | Ha | Alt1 | 800.45 | 14.7 | 822.1 | 24.2 | 0.065 |
| moderate effect |  | Alt2 | 1000.84 | 458.0 | 1001.1 | 53.8 | 0.003 |
| of frost on LAI |  | Alt3 | 1200.49 | 504.4 | 1202.2 | 55.2 | 0.000 |
|  |  | Alt4 | 1400.64 | 283.2 | 1378.0 | 57.3 | 0.033 |
|  |  | Alt5 | 1600.54 | 463.3 | 1596.4 | 55.5 | 0.004 |
| Evolution with | Hb | Alt1 | 800.45 | 26.8 | 820.4 | 24.2 | 0.054 |
| strong effect of |  | Alt2 | 1000.84 | 456.3 | 998.2 | 54.1 | 0.003 |
| frost on LAI |  | Alt3 | 1200.49 | 450.7 | 1193.6 | 54.3 | 0.001 |
|  |  | Alt4 | 1400.64 | 167.7 | 1359.3 | 46.0 | 0.049 |
|  |  | Alt5 | 1600.54 | 436.0 | 1593.9 | 54.0 | 0.005 |


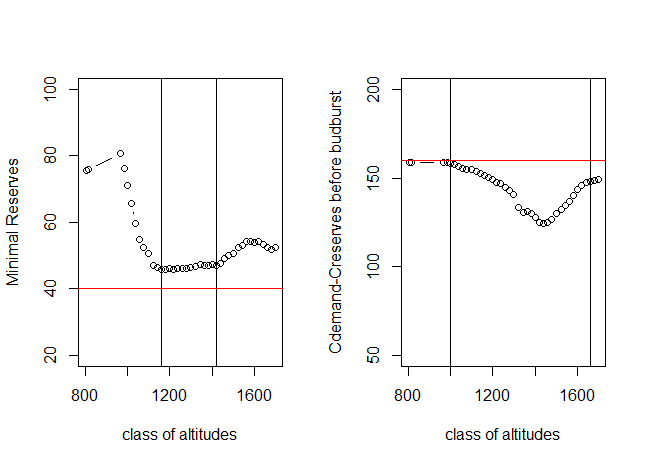


# Figure S1


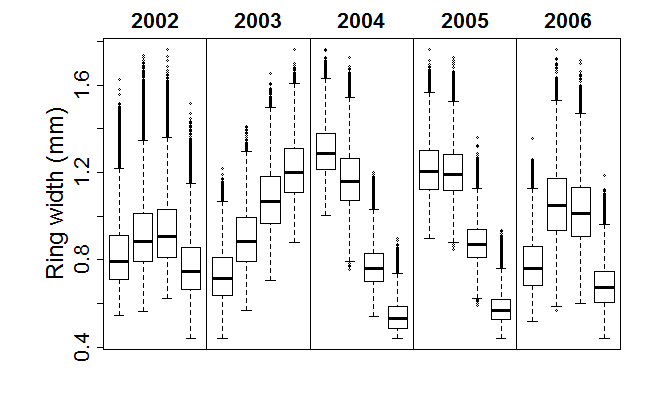

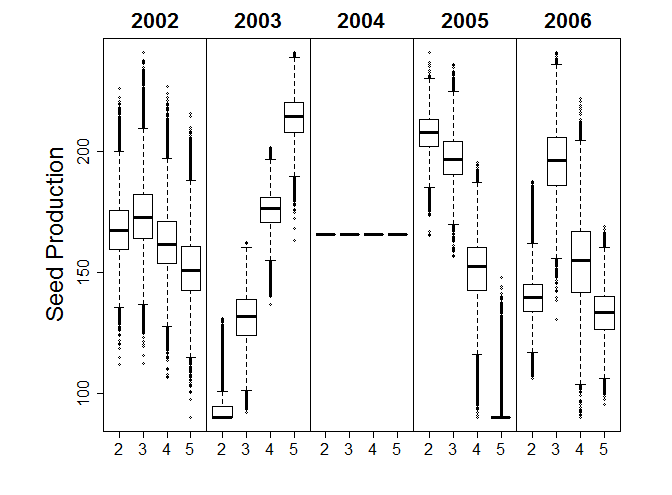

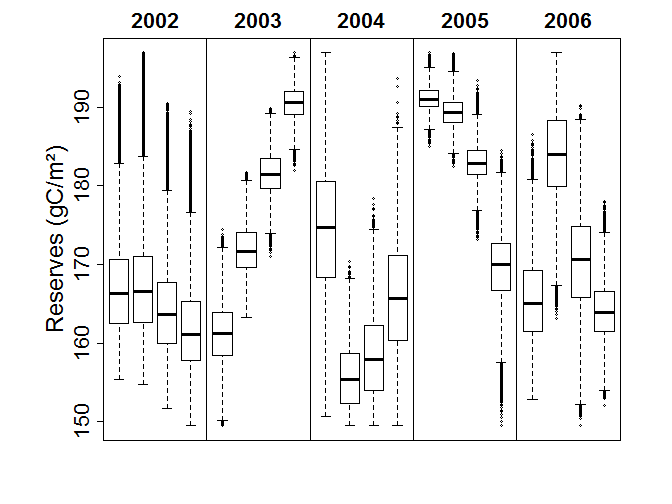

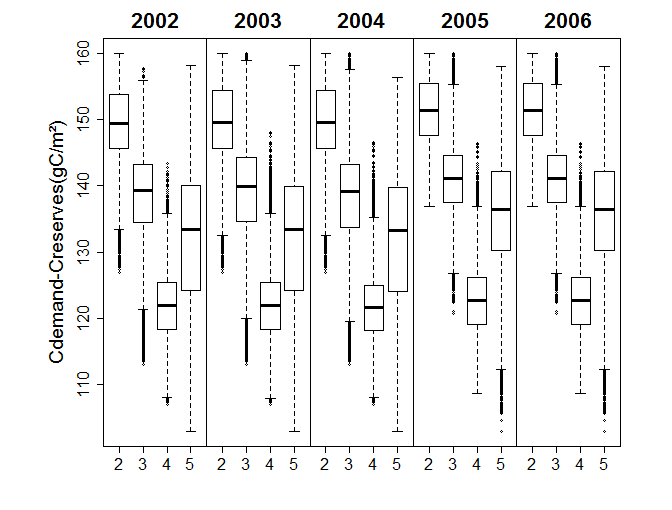


Population

A

B

Population

# Figure S2


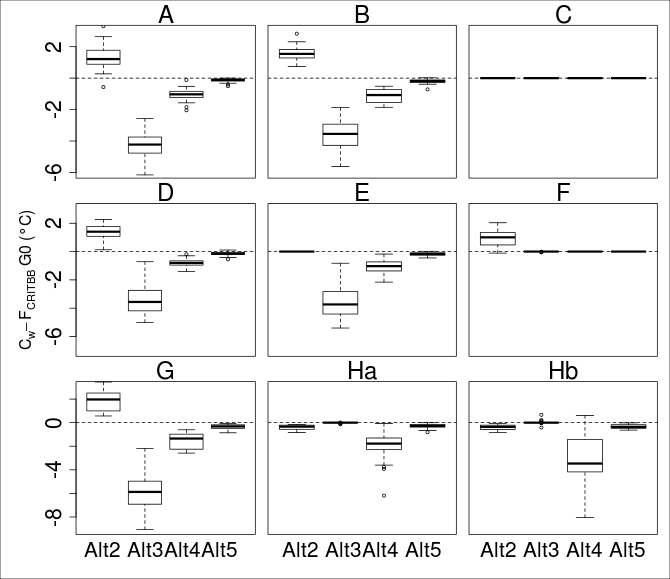


**Figure S3**


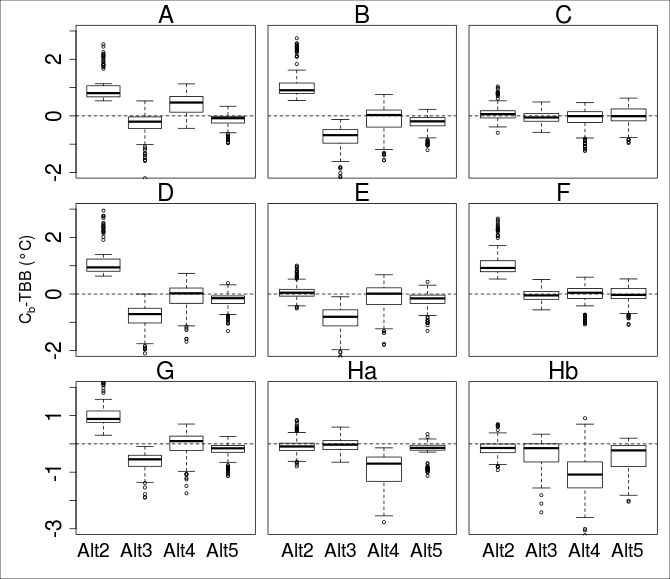


**Figure S4**
